# Different response of bacteria, archaea and fungi to process parameters in nine full‐scale anaerobic digesters

**DOI:** 10.1111/1751-7915.13409

**Published:** 2019-04-17

**Authors:** Susanne G. Langer, Christina Gabris, Daniel Einfalt, Bernd Wemheuer, Marian Kazda, Frank R. Bengelsdorf

**Affiliations:** ^1^ Institute of Microbiology and Biotechnology Ulm University Ulm Germany; ^2^ Institute of Systematic Botany and Ecology Ulm University Ulm Germany; ^3^ Genomic and Applied Microbiology & Göttingen Genomics Laboratory Georg‐August University Göttingen Göttingen Germany; ^4^ Present address: Bühlmann Laboratories AG Schönenbuch Switzerland; ^5^ Present address: Institute of Food Science and Biotechnology University of Hohenheim Stuttgart Germany

## Abstract

Biogas production is a biotechnological process realized by complex bacterial, archaeal and likely fungal communities. Their composition was assessed in nine full‐scale biogas plants with distinctly differing feedstock input and process parameters. This study investigated the actually active microbial community members by using a comprehensive sequencing approach based on ribosomal 16S and 28S rRNA fragments. The prevailing taxonomical units of each respective community were subsequently linked to process parameters. Ribosomal rRNA of bacteria, archaea and fungi, respectively, showed different compositions with respect to process parameters and supplied feedstocks: (i) bacterial communities were affected by the key factors temperature and ammonium concentration; (ii) composition of archaea was mainly related to process temperature; and (iii) relative abundance of fungi was linked to feedstocks supplied to the digesters. Anaerobic digesters with a high methane yield showed remarkably similar bacterial communities regarding identified taxonomic families. Although archaeal communities differed strongly on genus level from each other, the respective digesters still showed high methane yields. Functional redundancy of the archaeal communities may explain this effect. 28S rRNA sequences of fungi in all nine full‐scale anaerobic digesters were primarily classified as facultative anaerobic *Ascomycota* and *Basidiomycota*. Since the presence of ribosomal 28S rRNA indicates that fungi may be active in the biogas digesters, further research should be carried out to examine to which extent they are important players in anaerobic digestion processes.

## Introduction

Biogas is produced by complex anaerobic microbial communities, which degrade biomass to methane and carbon dioxide. An efficient biogas process is necessary to guarantee high degradation degree of organic compounds supplied with the feedstocks (volatile solids (VS) degradation), and consequently high methane yields. The reasons for differing performance between individual anaerobic digesters are often poorly understood (Werner *et al*., 2011). Besides process parameters and feedstock compositions, the efficiency of the biogas process depends on the interactions within the complex microbial network. Several studies showed by fluorescence‐based microscopic methods that anaerobic digesters contain about 10^10^ bacterial and 10^8^ methanogenic archaeal cells ml^−1^ (Krakat *et al*., 2010; Nettmann *et al*., 2010; Bengelsdorf *et al*., 2013; Langer *et al*., 2014). In general, bacteria contribute to the initial steps of biogas production: the hydrolysis of complex organic polymers, acidogenesis of organic monomers and acetogenesis of short‐chain fatty acids and alcohols. In the final step, methanogenic archaea convert acetate, some C_1_ compounds or carbon dioxide (CO_2_) and hydrogen (H_2_) to methane (Schink, 1997). Methanogens are either hydrogenotrophic (reduction of CO_2_ with H_2_ or formate), acetoclastic (splitting of acetate) or methylotrophic (reduction of methyl groups of methylated compounds e.g. methanol) and gain energy from methane production (Hedderich and Whitman, 2006; Dziewit *et al*., 2015). Both, hydrogenotrophic and acetoclastic methanogenic archaea have primarily been verified to be responsible for methane production during anaerobic digestion.

Lignocellulosic biomass has a high potential as feedstock for production of renewable energy, since it is the most common renewable resource on earth (Millati *et al*., 2011). Under anaerobic conditions, lignocellulosic biomass is persistent and only poorly degraded (Kazda *et al*., 2014). Hemicellulose and cellulose fractions are embedded in lignin (Lynd *et al*., 2002) which makes hemicellulose and cellulose not easily accessible for bacterial degradation. Fungi could open new possibilities for anaerobic digestion of lignocellulosic biomass (Kazda *et al*., 2014). A wide range of fungi has a high fibrolytic potential. Under aerobic and anaerobic conditions, various fungi degrade plant material very efficiently. Still, the presence and function of active fungi in anaerobic digesters have been barely investigated. Dollhofer *et al*. (2017) showed the presence of obligate anaerobic fungi (*Neocallimastigomycota*) in 7 of 10 agricultural biogas plants. Transcriptional cellulolytic activity of these fungi could only be detected in two of these biogas plants (Dollhofer *et al*., 2017). In general, members of *Neocallimastigomycota* have been observed also in the rumen of cows and the gastrointestinal tract of herbivore mammals and reptiles (Dollhofer *et al*., 2015). Here, the main function of for instance *Neocallimastix patriciarum* or *Piromyces* strain E is the degradation of lignocellulosic biomass (Teunissen *et al*., 1991). Besides enzymatic degradation (cellulosome or freely secreted enzymes), fungi can break plant material mechanically by their hyphae (Akin and Rigsby, 1987; Dollhofer *et al*., 2015). Thereafter, bacterial cells have access to plant tissues for further enzymatic degradation of cellulose and hemicellulose fractions. However, the study by Dollhofer *et al*. (2017) did not consider facultative anaerobic fungi. The presence of facultative anaerobic fungi in anaerobic digesters has been mainly investigated by means of DNA sequencing (Ritari *et al*., 2012; Bengelsdorf *et al*., 2013; Kazda *et al*., 2014). Young *et al*. (2018) identified and characterized lignocellulolytic aerobic and anaerobic fungi isolated from one‐ and two‐stage biogas plants. Members of the phyla *Ascomycota* and *Basidiomycota* could be identified in anaerobic digesters supplied with different feedstocks. As of yet, it is not clear whether these fungi were active in biomass degradation in the investigated digesters.

In contrast to fungal communities, bacterial and archaeal communities present in anaerobic digesters have been extensively analysed under various conditions by different molecular approaches based on 16S rDNA sequences and metagenome studies (Sundberg *et al*., 2013; Abendroth *et al*., 2015; De Vrieze *et al*., 2015; Klang *et al*., 2015; Langer *et al*., 2015; Westerholm *et al*., 2018). Nevertheless, the presence and microbial abundances based on DNA extracts do not necessarily reflect metabolic activity of these microbes (De Vrieze *et al*., 2016). This may lead to incorrect interpretation of community parameters. Community‐based analysis by means of extracted RNA can detect a stronger and faster response of the microbial assembly to environmental changes. Therefore, RNA‐based community screening can give more reliable information on actually active community members and to identify key species that determine the AD process (De Vrieze *et al*., 2016, 2018; Ziels *et al*., 2018). Same is true for studies dealing with metatranscriptomic approaches to investigate microbial community responses with respect to altered process conditions (Zakrzewski *et al*., 2012; Bremges *et al*., 2015; Güllert *et al*., 2016; Maus *et al*., 2016; Hassa *et al*., 2018).

The present study investigated nine anaerobic digesters from different biogas plants. Initially, anaerobic digesters were chosen without any preselection except for the willingness of the operator to cooperate. Specifications of these biogas plants and individual feedstock compositions were evaluated by questionnaire and have been already documented (Gabris *et al*., 2015; Einfalt and Kazda, 2016). Some biogas plants included more than one AD. Since those agricultural biogas plants were not equipped with analytical instruments to monitor each AD separately with respect to the amounts of biogas produced only one AD was sampled and process parameters recorded for the entire plant. Recorded process parameters included technical characteristics such as temperature, pH, organic loading rate, hydraulic retention time, degradation of volatile solids, specific biogas and methane production as well as feedstock composition. Moreover, chemical characteristics included the total amounts of solids, volatile solids, carbon, nitrogen, the ratio of volatile fatty acids to total inorganic carbon, ammonium, nitrate, phosphate, sulphate, ethanol, acetone, acetate, propionate and butyrate. Finally, for biological characteristics, the focus was set to microbial composition of bacterial, archaeal and fungal communities which were present in the nine agricultural anaerobic digesters. For this purpose, microbial communities were assessed by pyrotag sequencing of ribosomal RNA amplicons (16S and 28S rRNA) generated from environmental RNA by reverse transcription PCR. In that context, it has to be mentioned that analysing rRNA is not a general indicator of currently active microbes in environmental samples. But rRNA is an indicator of protein synthesis potential (Blazewicz *et al*., 2013). Active microbes are contributing to key metabolic processes during the anaerobic digestion process and do not necessarily perform constant cell division. Thus, RNA detection of a specific taxon indicates the potential of protein synthesis, and in that sense, the respective microbes are considered to be active.

Technical and chemical parameters were examined using Pearson correlation, similarities between microbial communities were compared using principal coordinates analysis (PCoA), and canonical correlation analysis (CCA) was performed to determine possible correlations between microbial community members and respective process parameters.

The underlying hypothesis was that the composition and structure of active microbial communities that provide high biogas yields are similar in the investigated anaerobic digesters. Moreover, major taxonomic groups assigned to the rRNA sequences were related to process parameters in order to test the (zero‐)hypothesis, that the taxa are not influenced significantly by the process parameters. Furthermore, we hypothesized that fungi are present and fungal ribosomal rRNA can be identified in the slurry samples. This could indicate that fungi are metabolically active and might be important in the anaerobic digestion process.

## Results

### Process parameters of nine anaerobic digesters

Slurry samples and process parameters were obtained from nine agricultural full‐scale anaerobic digesters located in southern Germany (Table 1). Studied digesters (abbreviated A–J) were operated under varying temperatures (17–49°C) at low (G and H: < 1 kg_VS_ m^−3^ d^−1^), moderate (1.11–2.33 kg_VS_ m^−3^ d^−1^) or high organic loading rates (OLRs) (B, 4.4 kg_VS_ m^−3^ d^−1^). Digesters G (25°C) and H (17°C) were run at low temperatures and showed low volatile solids (VS) degradation (G: 26%; H: 22%) in comparison with the other digesters (in average 69% VS degradation). The digesters A, C, D and I showed biogas yields of more than 500 l_N_ kg^−1^ VS and are termed high‐yield anaerobic digesters. Carbon/nitrogen (C/N) ratios ranged between 9.9 and 15.3, and methane percentage ranged between 53% and 57.6%. The volatile fatty acids to total inorganic carbon (VFA/TIC) ratio in the digester B (0.21) operated at the highest OLR was increased by factor 2 compared to other digesters (0.04–0.11). Moreover, the acetate levels were moderately elevated in digesters B (0.61 g l^−1^) and H (0.56 g l^−1^). Low specific methane production was positively correlated to both acetic (*R* ^2 ^= 0.87, *P* < 0.01) and propionic acid (*R* ^2 ^= 0.64, *P* < 0.05) concentrations. However, no further correlations were found between the analysed process parameters of the investigated anaerobic digesters. The ethanol concentration was increased in digester A (0.23 g l^−1^) compared to the other digesters (≤ 0.05 g l^−1^). Digesters G and H showed low ammonia levels (both 0.03 g l^−1^), digesters A, F and I had increased ammonia levels (0.53–0.86 g l^−1^), and the remaining digesters B, C and D exhibited critical levels of ammonia (> 1.2 g l^−1^). Further details regarding process parameters are listed in Table 1.

**Table 1:** Process parameters of studied anaerobic digesters

Anaerobic digester	A*	B	C*	D*	F	G	H	I*	J
Labeling of Einfalt and Kazda, (2016) a	8	nc^b^	11	13	nc	6	2	12	3
process temperature [°C]	41	40	44	46	42	25	17	43	49
Organic loading rate [kg_VS_ m^−3^ day^−1^]	2.33	4.40	1.21	2.05	2.08	0.90	0.95	1.92	1.11
Hydraulic retention time [day]	130	78	90	120	131	120	80	160	130
Degradation of VS [%] c	70.8	nda	64.9	63.9	nda	26.4	22.2	72.0	75.5
Specific biogas production [l_N_ kg^−1^ VS]	506	240	521	570	nda	434	382	549	445
Specific methane production [l_N_ kg^−1^ VS]	269	126	287	304	nda	236	209	294	256
Feedstock_FW_ [%]	cattle m. (74%)	maize s. (68%)	cattle m. (38%)	grass s. (32%)	maize s. (52%)	cattle m. (93%)	cattle m. (99%)	grass s. (43%)	pig m. (68%)
	grass s. (26%)	cattle m. (21%)	grass s. (32%)	crop s. (29%)	cattle m. (36%)	grass s. (5%)	straw (1%)	cattle m. (39%)	cattle m. (23%)
		chicken m. (11%)	grain s. (30%)	cattle dung (21%)	grass s. (11%)	cattle dung (2%)		cattle dung (18%)	fresh grass (4%)
				cattle m. (18%)		crop s. (1%)			grass s. (3%)
									crop debris (2%)
Methane [%]	53.1	53.0	55.0	53.3	nda	54.4	54.6	53.4	57.6
Total solids, TS [%]	10.2	10.9	8.7	12.5	7.2	10.1	6.6	8.5	8.8
Volatile solids, VS [%] (% of TS)	61.2	73.0	56.0	55.0	47.6	62.0	62.2	58.5	43.7
Nitrogen, N [%]	2.6	nda	3.1	2.5	3.4	2.6	2.6	3.1	2.5
Carbon, C [%]	39.8	nda	39.8	36.8	33.9	38.7	40.2	39.4	26.5
C/N	15.3	nda	13.0	14.6	9.9	14.8	14.5	12.9	10.6
pH	8.3	8.1	8.6	8.5	7.9	7.6	7.7	8.1	8.1
VFA/TIC	0.03	0.21	0.06	0.06	0.11	0.07	0.09	0.04	0.05
Ammonium, ${\text{NH}}_{4}^{+}$ [g l^−1^]	3.6	7.4	3.2	3.1	4.1	1.4	1.6	2.7	nda
Ammonia, NH_3_ [g l^−1^]	0.86	1.21	1.32	1.24	0.53	0.03	0.03	0.54	nda
Nitrate, NO^3−^ [mg l^−1^]	1.55	0.26	3.21	0.73	1.08	1.63	bdl	2.08	2.73
Phosphate, ${\text{PO}}_{4}^{3-}$ [mg l^−1^]	17.6	227	123	229	287	33.0	30.3	2.73	15.7
Sulfate, ${\text{SO}}_{4}^{2-}$ [mg l^−1^]	0.5	5.3	35	6.7	bdl	80	3.8	0.4	11.6
Ethanol [g l^−1^]	0.23	0.05	0.01	0.12	0.01	0.001	0.02	0.003	nda
Acetone [g l^−1^]	0.02	0.03	0.02	0.001	0.001	0.001	0.01	0.21	nda
Acetate [g l^−1^]	0.2	0.61	0.17	0.05	0.03	0.23	0.56	0.1	nda
Propionate [g l^−1^]	bdl	0.07	0.03	bdl	bdl	0.08	0.08	0.01	nda
Butyrate [g l^−1^]	bdl	0.08	0.006	bdl	bdl	0.02	0.008	0.02	nda

bdl, below detection limited; digester E was excluded from further analysis due to limited information regarding process parameters; m., manure; nda, no data available; s., silage; VS, volatile solids.

* High yield ADs (specific biogas yield >500 l_N_ kg^−1^ VS); nc, not included in the study of Einfalt and Kazda, (2016).

**a**. Gabris, C., Bengelsdorf, F.R., Dürre P. (2015) Analysis of the key enzymes of butyric and acetic acid fermentation in biogas reactors. *Microb Biotechnol* 8: 865–873.

**b**. Einfalt, D., Kazda, M. (2016) Characterisation of biogas plants on organic farms and potentials for improvement. *Org Agric* **6**: 243–2.

**c**. The calculated VS degradation was based on the difference of daily VS input of feedstock and VS in first reactor and is therefore lower compared to complete anaerobic digestion; VS, volatile solids; m., manure; s., silage; nda, no data available; bdl, below detection limited; digester E was excluded from further analysis due to limited information regarding process parameters

### Molecular assessment of microbial communities

Microbial communities of each anaerobic digester were investigated by prokaryotic 16S rRNA and fungal 28S rRNA pyrotag sequencing generated from cDNA obtained from total RNA extracts by reverse transcription (RT)‐PCR. PCR primers were used that amplified the V3‐V6 region of bacterial 16S rRNA fragments (approx. 760 bp) and the V4–V6 region of archaeal 16S rRNA fragments (approx. 550 bp). Regarding fungal 28S rRNA fragments, primers were used that target the highly variable region D1/D2 (approx. 650 bp). Subsequently, 454 pyrosequencing resulted in a total of 1 138 627 unprocessed 16S rRNA and 28S rRNA sequences which were analysed using QIIME (Quantitative Insight Into Microbial Ecology). The average sequence read length was 762 bp for bacterial, 561 bp for archaeal and 645 bp for fungal rRNA gene sequences respectively. Classification of 16S and 28S rRNA sequences, which were clustered as operational taxonomic units (OTUs, (sequence identity of 97%)), was done with an identity threshold of 90. On average, 94.8% of bacterial and 92.6% of archaeal sequences were assigned at phylum level, 90.9% and 87.0% sequences on family level as well as 74.5% and 86.7% sequences at genus level respectively. Furthermore, 59.1% of fungal sequences were assigned on phylum level, 58.9% sequences on family level and 57.1% sequences on genus level. Total numbers of sequences, observed OTUs, and Shannon index H′ values per sample obtained by the QIIME workflow (see Table S2) were used for further downstream analysis. Shannon index H′ (diversity index) for bacterial communities ranged between 3.9 and 5.5 (∅ H′ 4.8). Thus, they were more diverse in comparison with archaeal communities (∅ H′ 3.3, range: 1.7 to 4.6). Fungal communities showed the lowest diversity (∅ H′ 2.2), while the Shannon index ranged between 1.3 and 3.8.

### Composition of microbial communities

**Figure 1:**
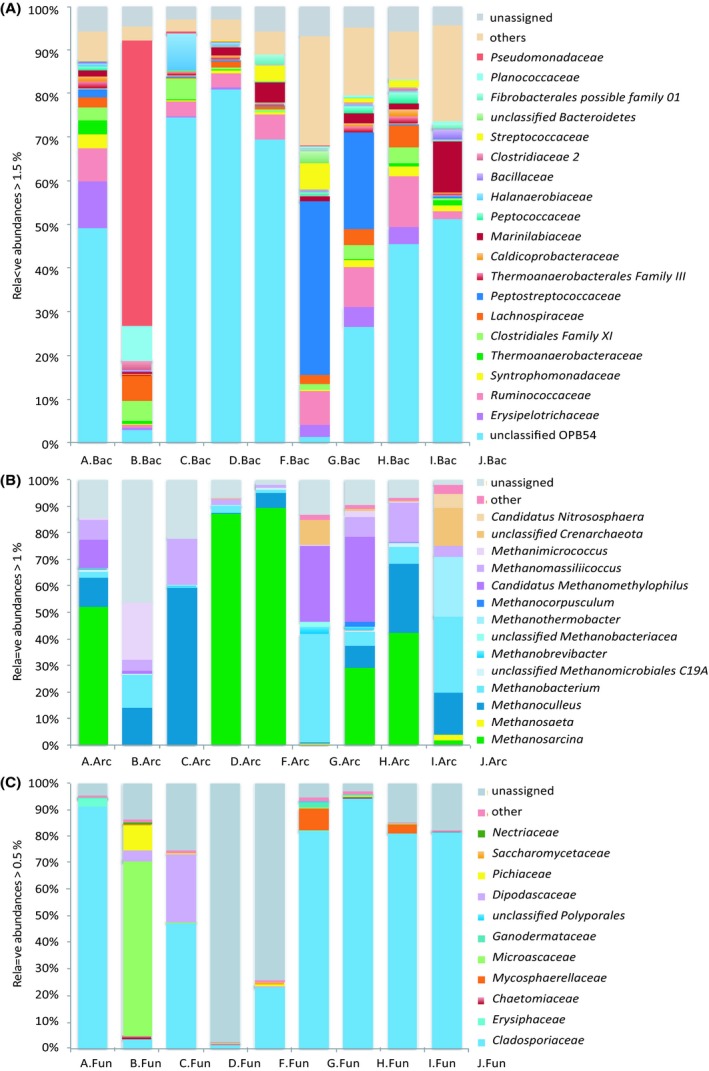
Composition of (A) bacterial, (B) archaeal and (C) fungal communities, based on rRNA analysis, involved in biogas production of the analysed anaerobic digesters. Taxonomic assignments of 454 pyrosequencing reads are based on SILVA taxonomy (90% sequence identity). Community compositions for bacteria and fungi are shown on family level and archaea on genus level.

16S and 28S rRNA sequences of metabolically active bacteria, archaea and fungi obtained after quality filtering were classified according to SILVA taxonomy (Release 119) based on operational taxonomic units (OTUs > 97% sequence identity) and displayed in a bar chart (Fig. 1). Phylogenetic depiction was set to family level for bacteria and fungi and to genus level for archaea, since sufficient resolution was found for those taxonomic ranks. Bacterial communities in anaerobic digesters A, C, D, F, I and J showed a similar composition (Fig. 1A). In all of these digesters, uncultured bacteria of the class *Clostridia* termed as ‘unclassified OPB54’ (46–81%) and members of the family *Ruminococcaceae* (2–12%) were present in high relative abundances. The composition of the microbial community in digester B supplied with chicken manure differed strongly from microbial communities in other digesters. Members of the family *Pseudomonadaceae* (65%) were predominant as well as members of *Planococcaceae* (8%), *Lachnospiraceae* (6%) and *Clostridiales* family XI (4%). Additionally, digesters G and H which were operated at low temperatures and mainly supplied with cattle manure (G: 93%; H: 99%) showed high abundances of members of the family *Peptostreptococcaceae* (G: 40%; H: 22%). Further, members of *Peptostreptococcaceae* (1.8%) were present in digester A, which was also supplied with a high amount of cattle manure (74%) but operated at a higher temperature (41°C) compared to reactors G (17°C) and H (25°C).

Archaeal communities (Fig. 1B) in anaerobic digesters were dominated by members of the phylum *Euryarchaeota*. Less abundant phyla were *Thaumarchaeota* and *Crenarchaeota*. Members of the genus *Methanosarcina* can be both acetoclastic and hydrogenotrophic and were predominant in digesters F (90%), D (87%), A (52%) and I (42%). In contrast to the digesters D and F, archaeal communities in A and I additionally contained significant proportions of members of hydrogenotrophic genera (*Methanoculleus* (A: 11%), *Methanobacterium* (I: 7%)) as well as methylotrophic methanogens (genus *Candidatus Methanomethylophilus* (A: 11%) and genus *Methanomassiliicoccus* (A: 7%, I: 15%)).

Digester C and the slightly thermophilic digester J were dominated by hydrogenotrophic methanogens. In contrast, digester B and the low‐temperature digesters G and H contained considerable proportions of both hydrogenotrophic and methylotrophic methanogens (Fig. 1B).

Members of the fungal communities (Fig. 1C) were primarily classified as *Ascomycota* (*Pezizomycotina* and *Saccharomycotina*), *Basidiomycota* (*Agaricomycotina*,* Pucciniomycotina* and *Ustilaginomycotina*) and *Mucoromycotina* as an early diverging fungal lineage. The community compositions of digesters A, G, H, I and J were very similar. Members of the family *Cladosporiaceae* were dominant in these digesters (H: 94%; A: 91%; G, I, J: all 81%). Other fungi in these digesters belonged to the families *Erysiphaceae* (A: 3%), *Mycosphaerellaceae* (G: 8%; I: 3%) and *Ganodermataceae* (G, H both < 2%). In digester C, the most abundant members belonged to the families *Cladosporiaceae* (47%) and *Dipodascaceae* (25%). In digester B, the community composed of members belonging to the families *Microascaceae* (66%), *Pichiaceae* (10%) and *Dipodascaceae* (4%). The majority of fungal OTUs of digesters D and F could not be assigned using the SILVA reference database. Therefore, representative sequences of those OTUs (> 500 sequences per OTU) were classified using BLASTn (NCBI nucleotide collection). That way, beforehand unassigned OTUs retained from digesters D and F were found to be related to up to now uncultured soil fungi. About 77% of all unassigned fungal sequences obtained from digester D clustered in two OTUs and showed highest similarity to a sequence annotated as ‘uncultured soil fungus clone OTU97‐71’ (NCBI accession no. JQ311505). Furthermore, 67% of unassigned sequences from digester F clustered also in a single OTU and showed highest identity to another 28S rRNA gene labelled as ‘uncultured soil fungus clone NCD_LSU_otu633’ (NCBI accession no. KF565603). Remaining sequences belonged to the families *Nectariaceae* (D: 1%) and *Cladosporiaceae* (F: 23%).

### Resemblance of microbial communities

**Figure 2:**
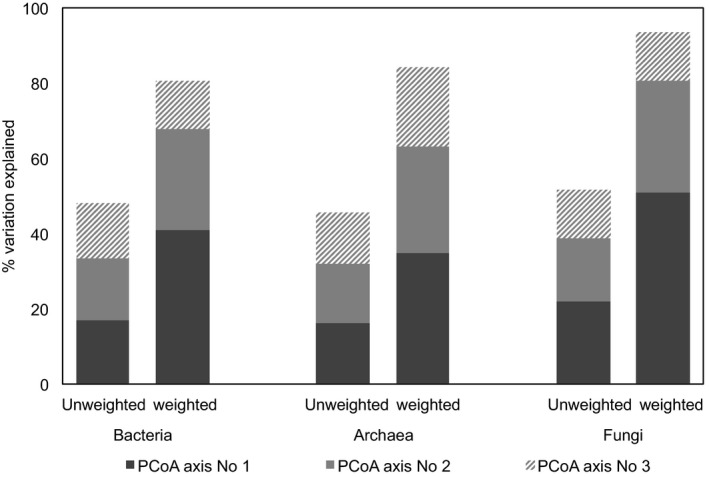
Variations explained by principal coordinate analysis (PCoA) axis PC1, PC2 and PC3. Dissimilarity matrices of bacterial, archaeal and fungal communities in nine different anaerobic digesters are based on unweighted and weighted UniFrac analysis. Unweighted UniFrac analysis poorly explains variations, whereas weighted UniFrac (includes abundances of taxa) data significantly increased the variations explained. Depth of coverage was adapted to the lowest number of reads.

Bacterial, archaeal or fungal community compositions were compared to each other by weighted (quantitative measures) and unweighted (qualitative measures) UniFrac analysis. Unweighted UniFrac analysis did not sufficiently explain variations between respective communities compared to weighted UniFrac analysis (Fig. 2). The weighted UniFrac algorithm incorporates the abundances of taxa and explained nearly twice as much of the variation between analysed communities in anaerobic digesters (Fig. 2).

**Figure 3:**
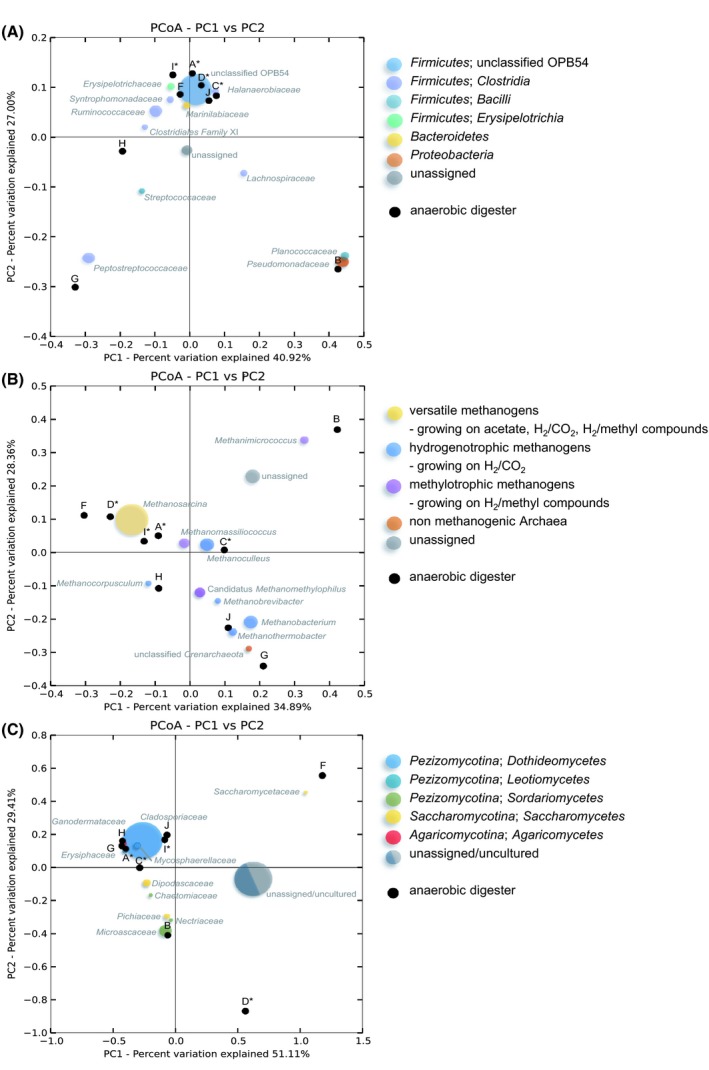
PCoA biplots of bacterial (A), archaeal (B) and fungal communities (C) based on phylogenetic distance matrices (beta‐diversity). Visualization was done using EMPeror. The distances of microbial communities were measured between pairs of samples by weighted UniFrac analysis (0, communities are identical). Biplots show clustering of microbial communities plotted along with abundances of the major taxa in the PCoA space. The size of each coloured dot represents the relative abundance of each of the taxa. Anaerobic digesters (black dots, * indicates high‐yield anaerobic digesters) ordinated closer to each other show a higher degree of similarity compared to communities displayed with increased distances. Moreover, the distance between coloured taxa dots and labelled anaerobic digesters expresses the distribution of taxa in the digesters.

Biplots were constructed from weighted UniFrac analysis metrics using principal coordinates analysis (PCoA) which displayed dissimilarities between microbial communities together with relative abundances of respective bacterial, archaeal or fungal taxa within communities (Fig. 3). Biplots for metabolically active bacterial communities (Fig. 3A) revealed high similarities between samples of digesters A, C, D, F, I and J. Bacterial communities in digesters G and H lined up along PCoA axis PC2 whereby the community in digester B differed in a similar way in respect to axis PC1. Operating conditions of the digesters G and H differed to the remaining digesters regarding temperature (G: 25°C; H: 17°C). Digester B was the only digester supplied with high amounts of chicken manure, but showed critical ammonia levels, which were in a similar range compared to those of digesters C and D (B: 1.21 g l^−1^, C: 1.32 g l^−1^, D: 1.24 g l^−1^). An outstanding feature of digester B regarding the operating conditions is the organic loading rate of 4.4 [kg_VS_ m^−3^ d^−1^], which is at least twice as high compared to the other digesters.

Archaeal communities in digesters A, D, F and I were dominated by versatile methanogens and clearly split along the PC1 axis. These communities were distinct from other communities that showed high abundances of hydrogenotrophic and/or methylotrophic methanogens (Fig. 3B). Moreover, this biplot showed that communities line up on the PC2 axis regarding the ammonia concentrations of the different digesters, whereas digesters B, C and D had the highest and digesters G and H had the lowest ammonia concentration. Archaeal communities of high‐yield digesters (A, C, D and I) differed in compositions as indicated by different distances to the PC1 axis (Fig. 3B).

The fungal communities of digesters A, C, G, H, I and J also showed a high degree of similarity (Fig. 3C). Low temperature obviously did not impact the fungal communities since digesters G and H clustered together with digesters operating at 42 to 49°C. Fungal communities of digesters B, D and F differed with respect to PCoA axis PC2. Fungi in digesters F and D were so far uncultured, and similar sequences have been previously detected in soils.

### Factors regulating microbial communities

Canonical correlation analysis (CCA) was applied to elucidate the relationship between process parameters or feedstocks and the respective microbial community composition. CCA was done separately for the four most abundant taxa of bacteria, archaea and fungi respectively. Within bacteria, the four most abundant groups were the ‘unclassified OPB54’, and the families *Ruminococcaceae, Peptostreptococcaceae* and *Pseudomonadaceae*. Their abundance showed best results for the process factors temperature, OLR and NH_4_^+^ concentration at high canonical *R* = 0.98 (*P* < 0.05). The first canonical root extracted about 69% of the variance within the process parameters and about 39% of the variance of the four most abundant bacterial groups. Within those, the abundance of the families *Ruminococcaceae* and *Peptostreptococcaceae* scaled in the opposite direction as the family of *Pseudomonadaceae. Pseudomonadaceae* increased with higher ammonium concentrations (*r* = 0.87) and the bacterial group OPB54 with higher ammonia (NH_3_) (*r* = 0.48). However, negative effects of NH_3_ were obvious for *Ruminococcaceae* and *Peptostreptococcaceae* (*r* = −0.74 and −0.77 respectively). Assessment of the influence of feedstock composition with higher amounts of cattle dung, cattle manure and maize silage on bacterial composition showed a significant effect (*P* < 0.01) but the variance extracted by the set of feedstocks was as low as 19%.

Relative abundance of four taxa of archaea (*Methanosarcina, Methanoculleus*, Candidatus *Methanomethylophilus* and *Methanomassiliicoccus*) showed close relationship to process parameters temperature, OLR and NH_4_^+^ concentration (canonical *R* = 0.98, *P* < 0.05). However, there was no significant influence of the parameter combination: temperature, OLR and ammonia (NH_3_) (*P* = 0.018). Temperature influenced negatively the abundance of Candidatus *Methanomethylophilus* (*R* ^2^ = 0.90, *P* < 0.05). The presence of the other two most abundant groups, *Methanoculleus* and *Methanomassiliicoccus,* was positively linked to each other (*R* ^2 ^= 0.77, *P* < 0.05). No significant influence on archaeal community composition was found regarding the used feedstocks (*P* = 0.18).

The abundances of fungi found in the investigated digesters showed no specific correlation to process variables (factor combination of temperature and OLR with NH_4_^+^ *P* = 0.34 and with NH_3_ *P* = 0.32 respectively). However, the first canonical root extracted about 41% of variance for the feedstocks and 45% for the presence of fungi respectively. Feedstocks cattle dung, cattle manure and maize silage were highly linked to the four most abundant fungi: *Cladosporiaceae, Microascaceae, Dipodascaceae* and the unassigned group (*P* < 0.01). Among those, *Cladosporiaceae* were positively correlated to cattle manure (*R* ^2 ^= 0.52, *P* < 0.05); the abundance of *Microascaceae* was linked to maize silage (*R* ^2^ = 0.66, *P* < 0.05).

## Discussion

Comparative studies of compositions of bacterial and archaeal communities in several (*n* ≥ 7) full‐scale anaerobic digesters were done based on extracted genomic DNA (Riviere *et al*., 2009; Sundberg *et al*., 2013; Abendroth *et al*., 2015; Li *et al*., 2015; Rui *et al*., 2015; Luo *et al*., 2016). In recent years, much more attention was paid to transcriptomic and proteomic approaches (for reviews see Heyer *et al*., 2016; Hassa *et al*., 2018). However, studies targeting the 16S rRNA of bacteria, archaea and 28S rRNA of fungi, respectively, are rare for anaerobic reactors (Lebuhn *et al*., 2014; De Vrieze *et al*., 2016; Dollhofer *et al*., 2017). The applied PCoA showed remarkable resemblances of the microbial communities in the analysed full‐scale anaerobic digesters (Fig. 3). In contrast to Caporaso *et al*. (2011), our results showed that PCoA on weighted UniFrac distance matrices (including abundances of taxa) was crucial in order to elucidate similarities of microbial communities. Sundberg *et al*. (2013) showed by principal component analysis that bacterial and archaeal communities were similar to each other in 14 biogas reactors digesting mixtures of different feedstocks but clustered separately from other 7 reactors digesting sewage sludge. Abendroth *et al*. (2015) investigated microbial communities of three different types of anaerobic digesters and found that each type showed highly similar communities.

### Bacterial communities and process parameters

Process conditions such temperature, organic loading rate as well as NH_3_ and NH_4_^+^ concentrations – as found in the present study – impacted primarily the bacterial communities, especially in the digesters B, G and H. Bacteria of the candidate taxon OPB54 (phylum *Firmicutes*) were found to be present in all investigated anaerobic digesters. Interestingly, remarkably high relative abundances of members of candidate taxon OPB54 (Fig. 1A) were found in digesters showing high biogas yields (A: 49.1%, C: 74.5%, D: 81.1%, and I: 45.5%). Within this taxon, only two OTUs were found to be dominant and several other OTUs showed minor abundances. This indicates that there were different species of the candidate taxon OPB54 present in anaerobic digesters. Hao *et al*. (2015) assumed that bacteria of this taxon may oxidize acetate and found that they were the most abundant bacteria in thermophilic, acetate‐fed batch experiments. Moreover, Li *et al*. (2015) found high abundances of bacteria identified as OPB54 in thermophilic solid‐state anaerobic batch reactors. However, the only isolated bacterium that belongs to the taxonomic group of OPB54 bacteria is *Hydrogenispora ethanolica* LX‐B^T^ (Liu *et al*., 2014). This bacterium is anaerobic, spore‐forming, produces ethanol and hydrogen using glucose as carbon source and was isolated from anaerobic sludge after herbicide wastewater treatment. Albeit different conditions have been tested, and *H. ethanolica* is not capable of a syntrophic metabolism with a partner that consumes hydrogen (Liu *et al*., 2014). Still it is tempting to speculate that OPB54 bacteria play a part in syntrophic acetate oxidation and were most probably promoting methanogenesis in high‐yield digesters as well as in digester J. As shown by CCA, digester B operating at high OLR combined with high NH_3_ concentration showed significantly fewer ribosomal rRNA fragments of candidate taxon OPB54 compared to the high‐yield digesters (Fig. 3A). Gabris *et al*. (2015) previously analysed this particular digester (B) (referred as BR1) for specific enzyme activities (acetate kinase, butyrate kinase and butyryl‐CoA:acetate‐CoA transferase), which were negatively correlated with ammonia and ammonium concentrations (Gabris *et al*., 2015). Moreover, in digester B high proportions of *Pseudomonadaceae* (*Proteobacteria*) were remarkable (65%). Members of the family *Pseudomonadaceae* have also been observed in other digesters with high ammonia concentrations (Su *et al*., 2015; Regueiro *et al*., 2016). There, respective bacteria probably contribute to methanogenesis via the syntrophic acetate‐oxidizing route (Regueiro *et al*., 2016). Surprisingly, ammonia concentrations based calculation (CCA) using temperature and pH values as parameters showed no significant influence on the composition of bacterial communities.

Further, bacteria such as members of the families *Clostridiales* family XI, *Erysipelotrichaceae, Ruminococcaceae*,* Lachnospiraceae* and *Syntrophomonadaceae* within the phylum *Firmicutes* are rather common in anaerobic digestion processes and accomplish various metabolic functions (Ziganshin *et al*., 2013; Langer *et al*., 2015; Li *et al*., 2015; Rui *et al*., 2015; Heyer *et al*., 2016).

CCA showed that *Peptostreptococcaceae* occurred mainly at low process temperatures (plants G and H) while the presence of *Pseudomonadaceae* was highly linked to OLR and NH_4_^+^ concentration (i.e. digester B). *Peptostreptococcaceae* belong to the *Clostridiales* within the phylum *Firmicutes*. *Peptostreptococcaceae* contain members which are able to grow under psychrophilic conditions, e.g. strains of *Proteocatella* spp. and *Tepidibacter* spp. (Slobodkin, 2014). Consequently, high abundance of *Peptostreptococcaceae* in digesters G and H can be due to an adaption of the microbial communities to these low temperatures. Moreover, *Peptostreptococcaceae* typically grow anaerobically via fermentation of proteinaceous feedstocks and some carbohydrates (Slobodkin, 2014). In those two digesters, the feedstock was composed of high amounts of cattle manure (G: 93%, H: 99%) and low amounts of plant‐derived feedstocks (Table 1). Thus, the feedstock composition with the high share of cattle manure seems to have caused the high abundance of *Peptostreptococcaceae*.

Some process parameters of the high‐yield digesters A, C, D and I differed from each other (e.g. OLR, feedstock composition, temperature). Nevertheless, bacterial community compositions in these high‐yield digesters as well as those in digesters F and J were similar to each other (Fig. 3A) and probably functioned in a similar way resulting in efficient biogas production. Bacterial communities in digesters G and H were shaped by the prevailing low‐temperature conditions and produced biogas at lower levels. Besides possible inhibition effects in the digester B, the other two high ammonia digesters C and D showed high specific methane yields (Table 1).

### Methylotrophy in archaeal communities

Archaea described to perform the acetoclastic, hydrogenotrophic or methylotrophic (H_2_‐dependent) methane production pathways were found to be present in varying proportions in all anaerobic digesters. Acetoclastic and hydrogenotrophic methanogens are well‐known contributors in the anaerobic digestion process. Methylotrophic archaea, that utilize methylated compounds (mono‐, di‐, tri‐methylamines or dimethylsulfide) as carbon source and hydrogen as energy source, are frequently reported as typical members of microbial biogas plant communities since their description (Chojnacka *et al*., 2015; Dziewit *et al*., 2015; Zhou *et al*., 2017). These methanogenic archaea have typically been found in digestive tracts and are also known to be present in human stool (Gorlas *et al*., 2012; Vanderhaeghen *et al*., 2015), human gut (Borrel *et al*., 2012; Gaci *et al*., 2014), sheep rumen (Li *et al*., 2016) and cattle rumen (Seedorf *et al*., 2015). The presence of ribosomal rRNA fragments of the genus Candidatus *Methanomethylophilus* and *Methanomassiliicoccus* within the characterized archaeal communities indicates that hydrogen‐dependent methylotrophy (Borrel *et al*., 2014) can contribute to methane generation in anaerobic digesters. This is reflected in the strong negative correlation found between the abundance of Candidatus *Methanomethylophilus sp*. and process temperature, which were mainly present in low‐temperature reactors G and H.

### Functional redundancy of the archaeal community

In high‐yield digesters (A, C, D and I), the archaeal community compositions differed from each other (Fig. 3B). Nevertheless, these communities performed their methane synthesis in similar rates albeit these differing compositions also imply different metabolic pathways. Therefore, these different communities can be considered as functionally redundant in respect of the last step in the methane production within the anaerobic digesters. Einfalt and Kazda (2016) also investigated seven of the nine anaerobic digesters represented in this study and examined fermentation characteristics and feedstock compositions. They found that high‐yield digesters (A, C, D and I) ran constantly and efficiently during a time course of nearly 2 years. Thus, functional redundancy is one of the most important features of the anaerobic digestion microbiome to maintain stable and efficient biogas production. Functional redundancy for archaeal communities has been previously shown only in laboratory‐scale anaerobic digesters (Langer *et al*., 2015; De Vrieze *et al*., 2017).

### Fungal communities and feedstock characteristics

There are only few reports about fungi in anaerobic reactors (Ritari *et al*., 2012; Bengelsdorf *et al*., 2013; Kazda *et al*., 2014; Dollhofer *et al*., 2017; Young *et al*., 2018). Fungal ribosomal rRNA from an astonishing variety of taxa (*Ascomycota* and *Basidiomycota*) was found in the present study. Remarkably, the presence of members of the phylum *Neocallimastigomycota* was not detected in the analysed anaerobic digesters, although all reactors were supplied with high proportions of cattle faeces (21–99%), and the presence of members of this phylum was previously linked to ruminants (Fliegerová *et al*., 2010; Dollhofer *et al*., 2015). The authors suggested that anaerobic fungi were presumably transported into the biogas digesters with the daily load of animal‐derived feedstocks.

In our study, the relative abundances of fungi were not linked to temperature and OLR neither in combination with NH_4_^+^ nor with NH_3_. However, the used feedstocks were of high significance. The members of *Cladosporiaceae* may have been brought into the reactor by cattle manure. On the other hand, the use of maize silage may have promoted the abundance of *Microascaceae*. Within the family of *Cladosporiaceae,* the genus *Cladosporium* is the most common (Bensch *et al*., 2012, 2015). There are approximately 170 *Cladosporium* species; however, only a small fraction is known from cultures (Bensch *et al*., 2012). Wirsel *et al*. (2002) analysed the substrate utilization patterns of 18 *Cladosporium* isolates from common reed (*Phragmites australis*) colonizing anaerobic wetland soils. These isolates grew on more than 60 different carbon sources, especially on various saccharides but showed only poor or no growth on carbonic and amino acids. It is tempting to speculate that detected *Cladosporiaceae* contribute to anaerobic digestion process.

*Microascaceae* spp. are common soil saprobes but information regarding their role in natural habitats is scarce (Lueders *et al*., 2006). Jirout *et al*. (2013) isolated *Pseudallescheria* spp. (family *Microascaceae*) from soil severely impacted by cattle. This soil showed patchy distributions of oxygen depletion indicating that *Pseudallescheria* spp. might be able to grow in micro‐aerobic or anaerobic environments. Moreover, Anastasi *et al*. (2005) isolated species of both families, *Microascaceae* and *Cladosporiaceae*, from compost (thermophilic 60°C, plant debris) and vermicompost (mesophilic 25–30°C) produced by the action of earthworms on plant and animal wastes after thermophilic preconditioning (60°C). *Microascaceae* spp. and *Cladosporiaceae* spp. showed the highest relative abundances in the investigated anaerobic digesters independently of process temperatures.

In digesters D and F, 28S rRNA gene sequences were identified which were similar to sequences previously found in two other studies (Eichorst and Kuske, 2012; Mueller *et al*., 2014). In both approaches, samples from soil depth between 10 and 30 cm from different sites were analysed using molecular techniques with respect to fungal communities degrading cellulose. Bengelsdorf *et al*. (2013) found by microscopy techniques that fungi accounted for 10^8^ cells ml^−1^ in a biogas reactor degrading food residues. Anaerobic digesters fed by fibrous plant materials may offer preferable living conditions for fungi, even without ruminant manure (Kazda *et al*., 2014). Therefore, fungi as significant player in the biomass degradation in various natural environments may also contribute to anaerobic degradation in biogas plants.

### Metabolic capabilities of fungi under anaerobic conditions

Fungi present in anaerobic digesters presumably degrade organic compounds to produce CO_2_ and ethanol in order to conserve energy. The most prominent example is the yeast *Saccharomyces cerevisiae*, which metabolizes glucose and other sugars by the Embden‐Meyerhof pathway (Lin and Tanaka, 2006). Additionally, there are some filamentous fungi conducting the biotransformation of cellulose to ethanol, e.g. *Monilia* sp. (family *Sclerotiniaceae*) (Saddler and Chan, 1982), *Neurospora crassa* (family *Sordariaceae*) (Gong *et al*., 1981) and *Peacilomyces* sp. (family *Trichocomaceae*) (Gervais and Sarrette, 1990). Members of the mentioned families were present in investigated anaerobic digesters, but only in minor abundances (< 0.5%). A further metabolic capability of fungi under anaerobic conditions is a metabolic process termed ammonia fermentation also called dissimilatory nitrate reduction to ammonia (DNRA) (An and Gardner, 2002; Jebaraj *et al*., 2010). Ammonia fermentation supports growth of fungi under ‘anoxic’ conditions (Zhou *et al*., 2002, 2010). In *Fusarium oxysporum,* probably an assimilatory NO_3_ reductase (aNar) and NO_2_ reductase (aNir) are used for reducing NO_3_^−^ to NH_4_^+^ using NAD(P)H functions as e^−^‐donor. For ammonia fermentation, this reaction has to be coupled with the catabolic oxidation of ethanol to acetate and substrate‐level phosphorylation (Zhou *et al*., 2002; Takasaki *et al*., 2004; Morozkina and Kurakov, 2007; Takaya, 2009). Fifteen of 17 fungal strains tested by Zhou *et al*. (2002) fermented ammonium under anaerobic conditions, suggesting that this activity is widely distributed among fungi. However, it is a conjecture that a biogas digester can provide suitable conditions for nitrate reduction as required for ammonia fermentation in distinct microhabitats within small spatial distance. Ammonia, nitrate, acetate and ethanol were present in various amounts in all investigated digesters. Thus, if ammonia fermentation by fungi is a feasible process under the highly reducing conditions in anaerobic digesters has to be validated experimentally.

## Experimental procedures

### Sampling and process parameters

Nine different agricultural anaerobic digesters, (A to J, ranging from 270 to 1600 m^3^ reactor volume) located in Baden‐Wuerttemberg and Bavaria, Germany, were sampled between September 2013 and January 2014 (Table 1). Detailed description and examination of fermentation characteristics concerning the digesters A, C, D, E, G, H, I, J and respective biogas plants is provided by Einfalt and Kazda (2016) (see Table 1 for cross‐references). Digester B is the main digester of the respective biogas plant. Further information regarding digester B is listed by Gabris *et al*. (2015). Digester F is the main digester of the respective plant. That specific plant operates with two completely stirred digesters in series. Samples were always drawn from the main digester regardless if the plant consisted of more than one digester in series. Prior to each sampling, digester content was stirred for about 10 min. Afterwards, digester content was drawn through a provided outlet, whereby the first litres were discarded. Subsequently, samples of each digester (3 × 200 g) were transported on ice in sealed plastic boxes (VDLUFA, 1983). Temperatures of samples were measured on site, whereas pH (Metrohm 605) and VFA/TIC ratios were immediately determined in the laboratory. Afterwards, all samples were examined for total solids (TS), VS, C, N, ammonium (NH_4_^+^), acetone, ethanol, acetate, propionate, butyrate, nitrate, phosphate and sulphate concentrations. Descriptions of standard analytical methods were specified in detail by Einfalt and Kazda (2016). Free ammonia concentrations were calculated according to the formula of Hansen *et al*. (1998).

### Nucleic acid extraction and cDNA synthesis

Total RNA from samples of anaerobic digesters (A–J) was extracted using the ZR Soil/Fecal RNA MicroPrep™ kit (Zymo Research Europe GmbH, Freiburg, Germany), following the manufacturer's instructions. Remaining DNA within RNA extracts was removed using the Turbo DNA‐*free*™ kit (Ambion^®^ by Thermo Fisher Scientific GmbH, Dreieich, Germany). The success of the DNase treatment was confirmed by standard polymerase chain reaction (PCR) on 16S and 28S rRNA gene fragments, which were visualized by gel electrophoresis. RNA concentrations were measured spectrophotometrically at 260 nm (NanoDrop 2000 Spectrophotometer, Thermo Fisher Scientific GmbH). For cDNA synthesis from total RNA, a standard reverse transcription (RT)‐PCR (Green and Sambrook, 2012) was performed using SuperScript™ III Reverse Transcriptase and random hexamer primers (Thermo Fisher Scientific GmbH). Approximately 200 ng of total RNA isolated from anaerobic digesters were used as template for each RT‐PCR reaction.

### Amplification and sequencing of 16S and 28S rDNA fragments

Primer sets and PCR conditions used for amplification of prokaryotic 16S rRNA, and fungal 28S rRNA are provided in Table S1. Barcode‐tagged primers (454 pyrosequencing approach) were used to amplify the V3–V6 region of bacterial 16S rRNA (760 bp) and the V4–V6 region of archaeal 16S rRNA (555 bp) from cDNA of samples by PCR as described previously (Langer *et al*., 2015) and listed in Table S1. Fungal 28S rRNA gene fragments were amplified from cDNA initially using primers (LR0R and LR3) without barcodes with a DNA‐free *Taq* DNA polymerase (AppliChem GmbH). These primers target the large subunit (LSU) and amplify the D1/D2 region (609 bp), which is the most variable region within the LSU gene (Porter and Golding, 2012). PCR fragments of the initial amplification served as templates for a second amplification using the same primers just tagged with respective barcodes. These PCR fragments were amplified by *ReproFast‐*DNA polymerase (Genaxxon Bioscience GmbH, Ulm, Germany) under slightly modified cycling conditions (denaturation step at 95°C and 15 cycles for each primer annealing temperature). For each sample (A–J), three independent PCR reactions were accomplished to generate bacterial, archaeal or fungal amplicons. Concentration of purified PCR fragments (DNA clean & concentrator™‐5 kit; ZYMO Research Europe GmbH) was measured using the NanoDrop 2000 Spectrophotometer at 260 nm. Finally, barcode‐tagged amplicons of bacterial 16S, archaeal 16S and fungal 28S rRNA fragments were equimolarly merged in separate pools. Sequences were determined by using pyrosequencing on Roche GS FLX++ sequencer with titanium chemistry (performed by MWG eurofins).

### Data analysis

Pyrosequencing data generated for bacteria, archaea and fungi were processed using QIIME (Caporaso *et al*., 2010a). Sequences were demultiplexed according to their specific barcodes. Sequences with a deviation greater than ± 10% from the expected fragment lengths were discarded as well as sequences with a quality score below 25 and homopolymer runs exceeding a limit of 6. Sequences were truncated at the first ambiguous ‘N’ character. The Uchime algorithm implemented in Usearch 6.0 was used in *de novo* mode (Edgar, 2010) to remove chimeric sequences. OTUs were also picked in the *de novo* mode based on 97% sequence similarity by Uclust (Edgar, 2010). The most abundant sequence was chosen as representative sequence for each OTU. Taxonomy was subsequently assigned to each OTU by Uclust alignment of representative sequences against the SILVA reference sequence database (Release 119) in QIIME. Sequences assigned to bacteria were removed from the archaeal data set. Furthermore, fungal sequences affiliated to prokaryotes or nonfungal eukaryotes were discarded. Representative sequences of fungal OTUs (> 500 sequences) that remained unassigned by using SILVA database were manually screened using BLASTn and the RefSeq NCBI database. Subsequently, sequences not related to any fungal sequence deposited in the RefSeq database were discarded. Furthermore, representative prokaryotic 16S rRNA gene sequences were aligned to a prealigned reference database (SILVA Release 119) using PyNAST (Caporaso *et al*., 2010b) and fungal 28S rRNA gene sequences were aligned using SINA 1.2.1.1 (Pruesse *et al*., 2012). Alignments were filtered, and phylogenetic trees were constructed by FastTree (Price *et al*., 2009). The processed data were used for α‐ and ß‐diversity measurements. Differences between microbial communities (ß‐diversity) were measured by unweighted and weighted UniFrac analysis (Lozupone *et al*., 2007) using QIIME. Principal coordinates analysis (PCoA) plots/biplots were generated by EMPeror based on weighted UniFrac analysis and relative abundances of the most common microbial taxa (Vázquez‐Baeza *et al*., 2013).

### Statistics

Pearson correlation coefficient *R* ^2^ was calculated for all process parameters given in Table 1 using Microsoft Excel 2011. CCA was done using the software Statistica ver. 6.1 [StatSoft (Europe) GmbH, Hamburg, Germany]. This evaluation can be applied to elucidate relationship between two lists of continuous variables. In the current study, this tool was used for process parameters (set one) and the microbial composition (set two) while each of the major domains, i.e. bacteria, archaea and fungi, was analysed separately. The four most abundant taxa were selected for the analysis from the respective domain (section: factors regulating microbial communities). The parameters process temperature, OLR, NH_3_ and NH_4_^+^ concentration were used as the other set of variables. Similarly, the importance of feedstocks (cattle dung, cattle manure and maize silage) for microbial composition was tested in a second CCA application.

### Accession number

Sequences were deposited in the EMBL database under the study accession number PRJEB11422.

## Conflict of Interest

The authors declare no conflict of interest.
